# Decreased Bone Mineral Density Is a Predictor of Poor Survival in Critically Ill Patients

**DOI:** 10.3390/jcm10163741

**Published:** 2021-08-23

**Authors:** Maximilian F. Schulze-Hagen, Christoph Roderburg, Theresa H. Wirtz, Markus S. Jördens, Lukas Bündgens, Samira Abu Jhaisha, Philipp Hohlstein, Jonathan F. Brozat, Philipp Bruners, Christina Loberg, Christiane Kuhl, Christian Trautwein, Frank Tacke, Tom Luedde, Sven H. Loosen, Alexander Koch

**Affiliations:** 1 Department of Diagnostic and Interventional Radiology, University Hospital RWTH Aachen, Pauwelsstraße 30, 52074 Aachen, Germany; pbruners@ukaachen.de (P.B.); ckuhl@ukaachen.de (C.K.); 2 Clinic for Gastroenterology, Hepatology and Infectious Diseases, University Hospital Düsseldorf, Medical Faculty of Heinrich Heine University Düsseldorf, 40225 Düsseldorf, Germany; christoph.roderburg@med.uni-duesseldorf.de (C.R.); markus.joerdens@med.uni-duesseldorf.de (M.S.J.); sven.loosen@med.uni-duesseldorf.de (S.H.L.); 3 Department of Medicine III, University Hospital RWTH Aachen, Pauwelsstraße 30, 52074 Aachen, Germany; thwirtz@ukaachen.de (T.H.W.); lbuendgens@ukaachen.de (L.B.); sabujhaisha@ukaachen.de (S.A.J.); phohlstein@ukaachen.de (P.H.); jbrozat@ukaachen.de (J.F.B.); ctrautwein@ukaachen.de (C.T.); tom.luedde@med.uni-duesseldorf.de (T.L.); akoch@ukaachen.de (A.K.); 4 Department of Diagnostic and Interventional Radiology, University Hospital Düsseldorf, Medical Faculty of Heinrich Heine University Düsseldorf, 40225 Düsseldorf, Germany; christina.loberg@med.uni-duesseldorf.de; 5 Department of Hepatology and Gastroenterology, Campus Virchow-Klinikum and Campus Charité Mitte, Charité Universitätsmedizin Berlin, Augustenburger Platz 1, 13353 Berlin, Germany; frank.tacke@charite.de

**Keywords:** osteoporosis, intensive care medicine, clinical/novel biomarkers, sepsis infections, survival

## Abstract

Alterations in bone mineral density (BMD) have been suggested as independent predictors of survival for several diseases. However, little is known about the role of BMD in the context of critical illness and intensive care medicine. We therefore evaluated the prognostic role of BMD in critically ill patients upon admission to an intensive care unit (ICU). Routine computed tomography (CT) scans of 153 patients were used to assess BMD in the first lumbar vertebra. Results were correlated with clinical data and outcomes. While median BMD was comparable between patients with and without sepsis, BMD was lower in patients with pre-existing arterial hypertension or chronic obstructive pulmonary disease. A low BMD upon ICU admission was significantly associated with impaired short-term ICU survival. Moreover, patients with baseline BMD < 122 HU had significantly impaired overall survival. The prognostic relevance of low BMD was confirmed in uni- and multivariate Cox-regression analyses including several clinicopathological parameters. In the present study, we describe a previously unrecognised association of individual BMD with short- and long-term outcomes in critically ill patients. Due to its easy accessibility in routine CT, BMD provides a novel prognostic tool to guide decision making in critically ill patients.

## 1. Introduction

Critical illness is defined as “any acute medical condition necessitating vital organ support without which death would be imminent” [1]. Despite intensive research efforts, the prediction of short- and long-term prognoses for critically ill patients has remained challenging [2]. Individual body composition was recently identified as a potential factor determining the outcome of patients treated in an ICU [3]. Besides sarcopenia, osteopenia, defined as the condition of abnormally low bone mineral density (BMD), has recently attracted increasing attention as a prognostic marker in the context of various clinical conditions [4,5,6]. Low BMD as well as resulting sequelae osteoporosis are commonly observed in aging societies; for example, in the United States, the prevalence of both conditions in patients >50 years of age is 44% and 10%, respectively [7]. The resulting economic consequences are significant, moreover, affected patients are at high risk for increased morbidity and mortality [8]. However, only very limited data on the relevance of BMD in critically ill patients exist to date.

The most commonly used tool for the diagnosis of low BMD is dual-energy X-ray absorptiometry (DXA). While the advantage of DXA is being a standardized and well-validated method of measurement, the obvious disadvantage arises from the fact that an additional examination must be performed, which is usually not possible in critically ill patients. CT scans of the thorax or abdomen are available in almost all patients treated on a medical ICU, offering the opportunity for opportunistic screening for low BMD in this vulnerable subgroup of patients [9,10].

By analysing a cohort of 153 critically ill patients treated on a medical ICU between 2006 and 2015, we aimed at examining whether a low BMD assessed by calculating the average voxel density within the first lumbar vertebra, might represent a prognostic factor in ICU patients.

## 2. Materials and Methods

### 2.1. Study Design and Patient Characteristics

This observational cohort study was performed to investigate the potential impact of bone mineralisation density (BMD) on the short- as well as long-term survival of critically ill patients who were admitted to a medical ICU. The study was approved by the local ethics committee (EK 150/06) of the University Hospital RWTH Aachen, Germany, and written informed consent was obtained from every participant or authorised relatives in case of unconsciousness.

### 2.2. Assessment of Vertebral Trabecular Attenuation at the First Lumbar Vertebra

**Figure 1:**
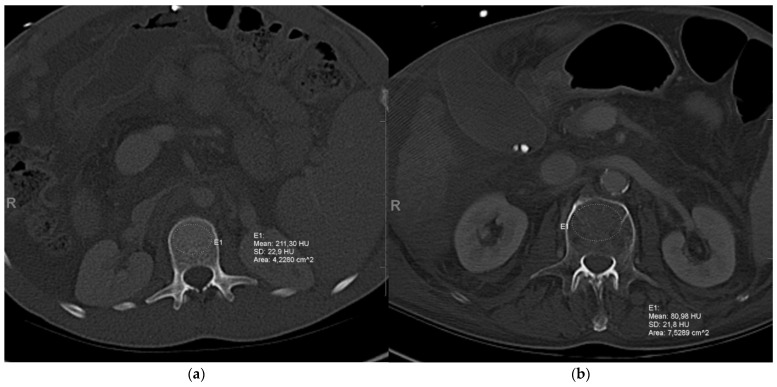
Assessment of bone mineral density (BMD) on routine CT scans. BMD was measured on routine CT scans upon ICU admission in the anterior portion of the trabecular space in the upper one-third of the L1. Two exemplary CT scans of patients with (**a**) high (221.3 HU) or (**b**) low BMD (80.98 HU) are shown.

We only included ICU patients with an available CT scan shortly before or after ICU admission into this study. The median duration between ICU admission and the CT scan was 1 day (IQR: 0–8 days). CT scans in soft tissue windows of 5 mm slice thickness were used for analyses. All measurements were performed in the local PACS (IntelliSpace PACS, Philips, Amsterdam, The Netherlands) by a consultant radiologist with 7 years of professional experience. An oval region of interest (ROI) was manually placed in the anterior portion of the trabecular space in the upper one-third of the L1, yielding the mean locoregional bone density in Hounsfield units (HU). The midvertebral level, as a zone of higher density, as well as the posterior trabecular space, with the venous plexus, were excluded to reduce measurement inheterogeneities. In addition, the ROI was adjusted to omit other potential confounders, such as osteomas, focal bone defects, or other bone tumours. The L1 was selected for measurements because it has been validated as a reference vertebral body in other studies and is commonly depicted in both chest CTs and abdominal CTs, respectively. If the L1 was fractured or a reasonable measurement could not be performed in this vertebral body, Th12 or L2 was used as an alternative [10]. Figure 1 exemplifies the bone density measurements in patients with high and low bone density.

### 2.3. Measurement of Standard Laboratory Parameters

All laboratory parameters were measured in the laboratory centre for blood analyses at University Hospital RWTH Aachen using a Sysmex XN9000 (Sysmex GmbH, Norderstedt, Germany) and Cobas 8000 c701 (Hoffmann-La Roche AG, Basel, Switzerland) platform. The following laboratory parameters were assessed: thrombocytes, INR, sodium, potassium, calcium, chloride, protein, albumin, cholesterol, bilirubin, AST, ALT, GGT, ALP, LDH, creatinine, urea, glucose, CK, CRP, PCT, IL-6.

### 2.4. Statistical Analysis

Shapiro–Wilk tests were performed to test for normal distribution of continuous data. Mann–Whitney U tests and Kruskal–Wallis tests were used to compare non-parametric data between two or more subgroups, respectively. ROC curves were generated by plotting sensitivity against 1-specificity. The predictive value of BMD regarding ICU survival was tested in binary logistic regression models. Kaplan–Meier curves were plotted to display impact on patients’ overall survival. Log–rank tests were performed to test for significant differences between subgroups. The prognostic value of variables was further tested by univariate and multivariate analyses in Cox regression models. Parameters with *p* < 0.05 in univariate analyses were included into multivariate analyses. Hazard ratios (HR) and 95% confidence intervals are displayed. All statistical analyses were performed with SPSS 23 (SPSS, Chicago, IL, USA). A *p*-value of <0.05 was considered statistically significant (* *p* < 0.05; ** *p* < 0.01; *** *p* < 0.001).

## 3. Results

### 3.1. Baseline Characteristics of the Study Cohort

In total, 153 patients admitted to the ICU of the Department of Medicine III at University Hospital RWTH Aachen between 2006 and 2015 were included into the study (patient characteristics are shown in Table 1). The median age of the study population was 60 years (range: 21 to 88 years). In total, 62.1% of patients were male and 37.9% were female. Sepsis with pulmonary focus (24.2%) was the most common diagnosis leading to ICU admission, followed by sepsis of other origin (15%), decompensation of cardiopulmonary disease (14.4%), abdominal sepsis (10.5%), decompensation of liver cirrhosis (7.8%), acute gastrointestinal bleeding (7.2%), acute pancreatitis (4.6%), acute liver failure (4.6%), urosepsis (3.9%), liver transplantation (2.6%) and others (5.2%). The median bone mineral density (BMD) of the study cohort was 152 HU (range: 54 to 277 HU). Table 1 provides a detailed overview on patient characteristics.

### 3.2. Bone Mineral Density (BMD) and Patient Characteristics

**Figure 2:**
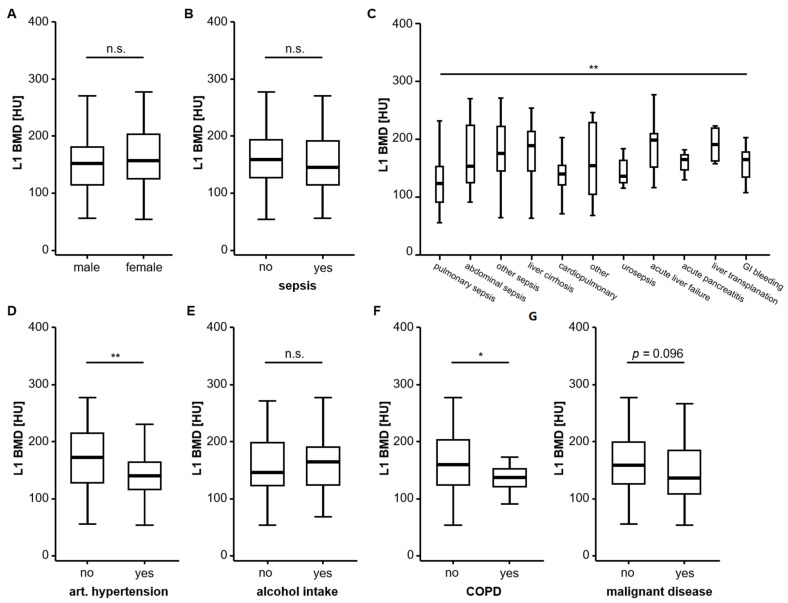
Bone mineral density (BMD) and patient characteristics. Male and female patients (**A**) and patients who did or did not fulfil the criteria of sepsis (**B**) have a comparable BMD. (**C**) BMD significantly differed between patients with different disease aetiologies that led to ICU admission. (**D**) BMD is significantly lower in patients with pre-existing arterial hypertension but comparable between patients with or without chronic alcohol intake (**E**). Patients with pre-existing chronic obstructive pulmonary disease (COPD) have significantly lower BMD values (**F**) and patients with pre-existing malignant disease show a strong trend towards lower BMD values (**G**). *: *p* < 0.05, **: *p* < 0.01.

To better understand the regulatory mechanisms of BMD in critically ill patients, we first evaluated a potential association between BMI and various clinicopathological parameters. While male and female patients (Figure 2A), as well as patients who did or did not fulfil the criteria of sepsis (Figure 2B), had a comparable BMD, we observed significant differences in the BMD between patients with different disease aetiologies that led to ICU admission (Figure 2C). As such, the median BMD was lowest among patients with pulmonary sepsis and highest in patients with decompensated liver cirrhosis or acute liver failure (Figure 2C). In addition, BMD was significantly lower in patients with pre-existing arterial hypertension (Figure 2D) but comparable between patients with or without chronic alcohol intake (Figure 2E). Finally, patients with pre-existing COPD had significantly lower BMD values (Figure 2F) and patients with pre-existing malignant disease showed a strong trend towards lower BMD values (*p* = 0.096, Figure 2G).

Subsequently, we performed correlation analyses between BMD and various standard laboratory markers to further identify potential drivers of BMD alterations (Table 2). Here, we observed a significant positive correlation between BMD and various parameters of liver function (bilirubin, AST, ALT, GGT, ALP), creatinine kinase and international normalized ratio (INR). In contrast, baseline BMD values negatively correlated with different serum electrolytes (sodium, potassium, chloride) as well as thrombocytes, blood urea and glucose levels (Table 2).

### 3.3. Low BMD Is Associated with Higher Short-Term Mortality in Critically Ill Patients

**Figure 3:**
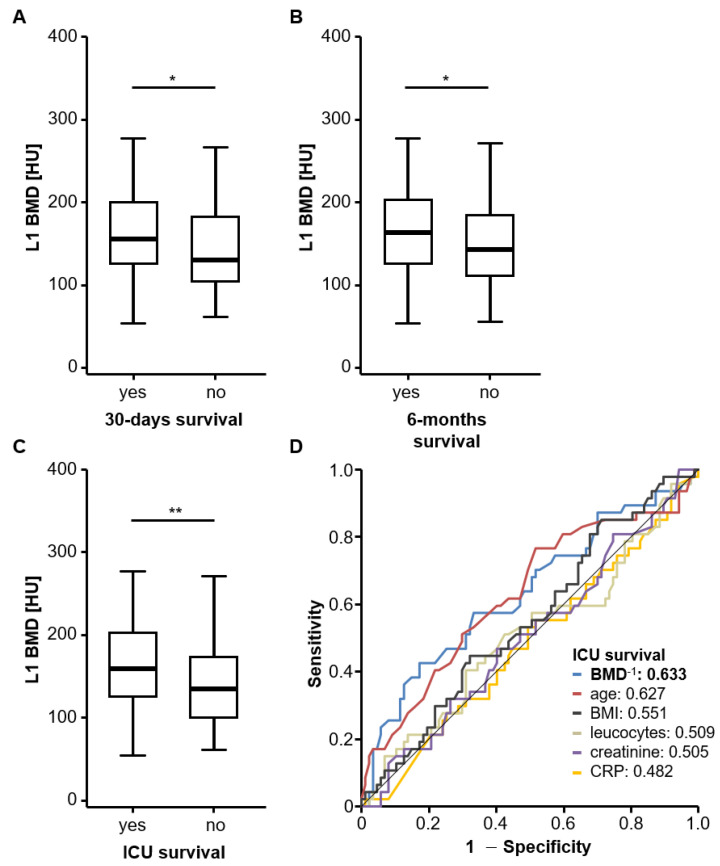
Low BMD is associated with higher short-term mortality in critically ill patients. (**A**) BMD values are significantly lower in ICU patients who died within the first 30 days after ICU admission. (**B**) BMD is significantly higher in patients who survived for six months following ICU admission compared to critically ill patients who died during this time period. (**C**) ICU patients who died on the ICU have significantly lower BMD values compared to patients who were discharged from the ICU. (**D**) ROC curve analysis reveals that BMD at ICU admission had an AUC value of 0.633 regarding the discrimination between ICU survivors and non-survivors, which was numerically higher than other known predictive factors for ICU survival. *: *p* < 0.05, **: *p* < 0.01.

Based on our hypothesis that low BMD might be associated with impaired outcomes in critically ill patients treated on an ICU, we next compared BMD values between patients who did survive the first 30 days following ICU admission and patients who died within this period. Interestingly, we observed significantly lower BMD values in patients who died within the first 30 days after ICU admission (Figure 3A). In line with this, BMD was significantly higher in patients who survived for six months following ICU admission compared to critically ill patients who died during this time period (Figure 3B). This observation was also confirmed with respect to ICU survival; patients who died on the ICU had a significantly lower median BMD of 134 HU compared to 159 HU in patients who were eventually discharged from ICU (Figure 3C). ROC curve analysis revealed that BMD at ICU admission had an AUC value of 0.633 regarding the discrimination between ICU survivors and non-survivors, which was numerically higher than other known predictive factors for ICU survival such as patient age (AUC: 0.627), BMI (AUC: 0.551) or laboratory markers of inflammation and kidney dysfunction (AUC_leucocytes_: 0.509, AUC_creatinine_: 0.505, AUC_CRP_: 0.482, Figure 3D). However, no statistically significant difference was revealed for these numerical differences found in the ROC analysis. At an optimal predictive cut-off value for ICU survival (121.5 HU), BMD showed a sensitivity and specificity of 83.9% and 42.9% for the prediction of ICU survival. In univariate logistic regression analysis, BMD upon ICU admission turned out to be a significant predictive factor for ICU survival (OR: 0.991, 95%CI: 0.984–0.998, *p* = 0.014).

### 3.4. BMD Is a Prognostic Factor for Overall Survival in Critically Ill Patients

**Figure 4:**
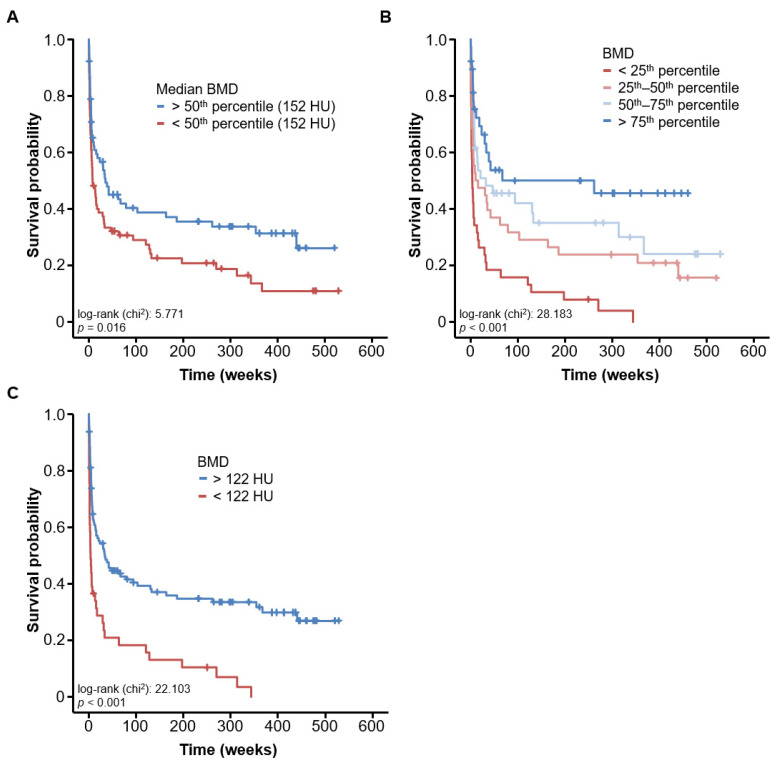
BMD is a prognostic factor for overall survival in critically ill patients. (**A**) ICU patients with a baseline BMD value < 152 HU have significantly impaired OS compared to patients with a BMD value above 152 HU. (**B**) Kaplan–Meier curve analysis reveals that OS is particularly poor in patients with BMD values below the 25th percentile. In these patients, median OS is only 3.29 weeks compared to 261.57 weeks in patients whose BMD upon ICU admission ranged within the upper quartile. (**C**) When applying the optimal prognostic cut-off value of 122 HU, Kaplan–Meier curve analysis shows significantly shorter OS in patients with a BMD value < 122 HU compared to patients with a BMD value above this cut-off value.

Based on the promising results regarding low BMD and impaired short-term outcomes in critically ill patients, we next hypothesized that low BMD might also be associated with impaired overall survival (OS). Overall, 73.2% of ICU patients died during follow-up. Median OS of the study cohort was 17.14 months. We first compared OS between patients with a BMD value above or below the cohort’s median BMD (152 HU). Interestingly, we observed that patients with a baseline BMD value < 152 HU had significantly impaired OS compared to patients with a BMD value above 152 HU (Figure 4A). When comparing the outcome of ICU patients stratified for the BMD quartiles of the entire cohort, Kaplan–Meier curve analysis revealed that OS was particularly poor in patients with BMD values below the 25th percentile (Figure 4B). In these patients, median OS was only 3.29 weeks compared to 261.57 weeks in patients whose BMD upon ICU admission ranged within the upper quartile (Figure 4B). Finally, we determined an ideal prognostic cut-off value that best discriminated between survivors and non-survivors as recently described. When applying this optimal cut-off value of 122 HU, Kaplan–Meier curve analysis showed a highly significantly shorter OS in patients with a BMD value < 122 HU compared to patients with a BMD value above this cut-off value.

In a next step, we performed uni- and multivariate Cox regression analyses to further corroborate the prognostic value of the patients’ BMD value at ICU admission and to identify potential confounders. In univariate analyses, a BMD value below the optimal cut-off value was a strong predictor of impaired OS (HR: 2.493, 95%CI: 1.679–3.701, *p* < 0.001). We further identified the patients’ age, serum potassium, AST, ALP, IL-6 and haemoglobin values as significant prognostic factors in univariate analyses and subsequently included these parameters into multivariate testing. Importantly, multivariate Cox regression analysis revealed that the prognostic value of a baseline BMD value below 122 HU was an independent predictor for impaired OS (HR: 1.836, 95%CI: 1.118–3.015, *p* = 0.016). Table 3 provides a detailed overview on Cox regression analyses.

## 4. Discussion

Prediction of outcomes is essential to guide interdisciplinary treatment decisions in critically ill patients. However, available tools including lab parameters of inflammation or organ dysfunction, as well as clinical scores, reflect only limited aspects of pathophysiology in critically ill patients and are of limited use in making prognostic and/or predictive decisions. Therefore, the ongoing search for novel predictive tools and algorithms is important to further improve treatment for this vulnerable group of patients. In the present manuscript, we identified the bone mineral density (BMD), which was assessed using routine CT scans upon ICU admission as a novel predictor of short- and long-term survival in critically ill patients treated on a medical ICU.

The concept of using routine CT scans to determine patients’ individual BMD is gaining increasing attention. A recent study including over 20,000 patients established age-corrected, normative values for CT graphically measured vertebral trabecular attenuation values (in Hounsfield units: HU) at the level of the first lumbar vertebra (L1) in patients aged 30–90 years [10]. BMD was assessed by calculating average voxel density within a ROI of a thoracic or lumbar vertebra (e.g., Th11 or L1), using CT images routinely available in the clinic. Since computed tomography of the thorax and abdomen is routinely performed at admission or during the early clinical course of ICU patients, osteopenia can easily be determined in these patients. In any case, our results integrate perfectly with recent efforts to use accruing imaging for additional purposes (opportunistic imaging, [11]).

Reduction of BMD is an increasingly important phenomenon, especially in an aging society, and the medical consequences are profound. It has already been shown that low BMD is associated with various conditions and diseases like cardiovascular diseases, stroke or chronic lung diseases [12,13,14]. However, it had so far remained unclear if low BMD has a prognostic value in ICU patients with different disease aetiologies. Our study results show that there was no significant difference in BMD with respect to patient demographics or distribution between patients with and without sepsis. In contrast, significant differences in BMD were found among patients with different disease aetiologies. In particular, patients with pulmonary sepsis as well as COPD showed the lowest BMD, while patients with non-infectious aetiologies of critical illness displayed rather high BMD. Notably, these findings are supported by the Rotterdam study by Campos-Obando and colleagues [13] and by results from Qu et al. showing that patients with chronic lung disease and arterial hypertension had also significantly decreased BMD [15].

In addition to differences in BMD with respect to disease aetiology, we also observed a strong association between BMD and patient outcomes both for short- and long-term mortality. In terms of short-term mortality, both 30 days survival, defined from the day of ICU admission, as well as ICU mortality were significantly worse in patients with low BMD compared to other patients. According to the above-mentioned study by Jang et al., the age-corrected normative value for the median patient age of our cohort (60 years) is 157 HU (SD: 38 HU). This coincides well with the cut-off value of 152 HU which we identified to allow discrimination of overall survival [10]. Correspondingly, the AUC regarding the distinction of ICU survivors to non-survivors based on BMD yielded 0.633, which was higher than all other outcome parameters such as patient age, BMI, leukocytes, creatinine and CRP; however, due to the minor variation in AUC values, no significant difference could be detected. In this respect, an optimal predictive value of BMD was found at a cut-off of 122 HU with a sensitivity and specificity of 83.9% and 42.9%, respectively. It must be noted that while the sensitivity is acceptable, the specificity must be deemed to be quite low. A limiting confounder with regard to ICU survival is the heterogeneity of disease distribution in the cohort, which might have played a major role in this context. Differences in BMD were also found regarding long-term survival. Kaplan–Meier curves showed that patients in the lower 25th percentile of BMD in particular had a severely shortened OS (lower quartile: 3.29 weeks vs. upper quartile: 261.6 weeks). When we applied the ideal cut-off value for OS (122 HU), we found a HR of 2.493 (95%CI: 1.679–3.701, *p* < 0.001) for the prediction of OS in Cox regression analysis. Importantly, the prognostic value of BMD was independent of several clinically relevant factors in multivariate Cox regression analysis. These results are in line with other studies that identified patients with reduced BMD as prone to impaired outcomes after trauma [16] as well as after major abdominal surgery [5].

The underlying cause of the correlation between low BMD and inferior outcomes in patients with critical illness remains unclear. One possible factor could be the connection between low BMD (as an indicator for osteoporosis) and an increased cardiovascular risk profile. These seemingly unrelated diseases might interact through common metabolic mechanisms, involving bone morphogenetic proteins, parathyroid hormone, phosphate, oxidized lipids, the RANKL-RANK-OPG pathway, MGP and vitamin D and K [15]. As a consequence, vascular calcifications are more frequent in patients with BMD, which is a known risk factor for unfavourable clinical course in ICU patients [16]. In addition, osteoporosis was also found to be an associated condition in patients with COPD [17] and chronic liver disease [18]. Here, too, the underlying pathomechanisms are not yet fully understood. Long-term administration of glucocorticoids in pulmonary disease and multifactorial inhibitory influences on osteoblastic cell function (sclerostin, retained substances of cholestasis, alcohol) are suspected. While the underlying causes of potential interactions between sepsis and osteoporosis have not yet been established, a recently published study demonstrated an association between these two conditions in younger patients in particular (<65 years of age) in a national study cohort of >13,000 patients in Taiwan [19]. These findings coincide well with our results.

Based on our findings, a perspective for subsequent research is to automate the process of bone density measurement in cross-sectional imaging of ICU patients and to establish an accurate model in a larger study cohort that estimates patient-specific risk for morbidity and mortality.

There are some limitations to this study. First, this is a retrospective single-centre study with a limited number of patients. Confirmation in a multicentre study with a higher number of cases is therefore required. Second, the heterogeneity of the included aetiologies of critical illness could lead to a bias of results. On the flipside, our cohort represents a realistic cross-section of disease distribution on a medical ICU of a large university hospital. Moreover, the heterogeneity of aetiologies argues for an aetiology-independent function of BMD as a prognostic marker. The significant correlations we observed between BMD and various laboratory markers are characterized by rather small correlation coefficients, limiting their clinical value and warranting further analysis to fully dissect the associations between BMD and these parameters. In addition, the current study does not include a control population, e.g., healthy volunteers or patients admitted to a standard care ward, which could yield important information on the influence of BMD on patient outcomes beyond the ICU setting. Despite the availability of the above-mentioned normative values found in a large study cohort of 20,000 patients, we consider this fact to be a significant limitation that should be addressed in future studies. Nevertheless, our study is the first of its kind to examine bone density in patients with critical illness and supports the concept of harvesting already available, otherwise underutilized data in terms of opportunistic imaging for early clinical decision making.

## 5. Conclusions

In conclusion, our study demonstrates that low BMD is a serious risk factor for patients with critical illness. Opportunistic imaging is a simple way to assess BMD in intensive care patients and allows a crude estimation of prognosis. Yet, multicentre studies with larger numbers of cases are needed to confirm our findings.

## Figures and Tables

**Table 1:** Characteristics of study cohort. BMI: body mass index, COPD: chronic obstructive pulmonary disease, GI: gastrointestinal, ICU: intensive care unit, HU: Hounsfield unit.

Parameter	Study Cohort
ICU patients	153
Gender (%) male-female	62.1–37.9
Age (years, median and range)	60 (21–88)
BMI (kg/m^2^, median and range)	25.21 (13.89–69.92)
Bone mineral density (HU, median and range)	152.0 (54.0–277.0)
ICU stay (days, median and range)	12 (1–399)
Hospital stay (days, median and range)	35 (3–199)
Sepsis (%) no–yes	38.8–61.2
Cause of ICU admission (%)	
Pulmonary sepsis	24.2
Abdominal sepsis	10.5
Urosepsis	3.9
Other sepsis	15
Liver cirrhosis	7.8
Acute liver failure	4.6
Liver transplantation	2.6
GI bleeding	7.2
Cardiopulmonal	14.4
Acute pancreatitis	4.6
Other	5.2
Medical conditions	
Diabetes mellitus	76.00%
Art. hypertension	50.40%
Coronary artery disease	75.20%
Chronic alcohol intake	69.50%
COPD	83.70%
Liver cirrhosis	73.60%
Malignant disease	68.20%
Survival rates	
ICU survival	68.20%
30-days survival	71.20%
180-days survival	43.10%

**Table 2:** Correlation analysis between BMD and various laboratory parameters.

Laboratory Parameters	BMD	
	Correlation Coefficient (r_S_)	*p*-Value
Leukocytes	−0.054	0.515
Haemoglobin	−0.087	0.289
Thrombocytes	−0.201	0.014
INR	0.177	0.030
Sodium	−0.269	0.001
Potassium	−0.161	0.049
Calcium	−0.141	0.087
Chloride	−0.277	0.001
Protein	0.104	0.252
Albumin	−0.006	0.953
Cholesterol	−0.102	0.267
Bilirubin	0.256	0.002
AST	0.389	<0.001
ALT	0.419	<0.001
GGT	0.260	0.001
ALP	0.180	0.034
LDH	0.291	0.001
Creatinine	−0.060	0.458
Urea	−0.211	0.009
Glucose	−0.194	0.025
CK	0.241	0.004
CRP	0.001	0.987
PCT	0.085	0.362
IL-6	0.074	0.415

BMD: bone mineral density, INR: International normalized ratio, AST: aspartate transaminase, ALT: alanine transaminase, GGT: γ-Glutamyl transpeptidase, ALP: alkaline phosphatase, LDH: lactate dehydrogenase, CK: creatinine kinase, CRP: C-reactive protein, PCT: procalcitonin, IL: interleukin, r_S_: Spearman’s correlation coefficient.

**Table 3:** Cox regression analyses including individual BMD and other clinicopathological parameters for the prediction of overall survival.

	Univariate Cox Regression		Multivariate Cox Regression	
Parameter	*p*-Value	Hazard-Ratio (95% CI)	*p*-Value	Hazard-Ratio (95% CI)
BMD < 122 HU	<0.001	2.493 (1.679–3.701)	0.016	1.836 (1.118–3.015)
Age	<0.001	1.030 (1.015–1.046)	0.002	1.034 (1.013–1.055)
Sex	0.724	0.933 (0.634–1.373)		
BMI	0.875	1.002 (0.976–1.029)		
Sodium	0.890	1.002 (0.976–1.028)		
Potassium	0.014	1.317 (1.057–1.642)	0.300	1.152 (0.881–1.506)
Calcium	0.919	0.959 (0.427–2.152)		
Leukocytes	0.386	1.008 (0.990–1.027)		
Hemoglobin	0.025	0.991 (0.983–0.999)	0.403	0.996 (0.986–1.006)
Platelets	0.616	1.000 (0.999–1.002)		
Protein	0.113	0.993 (0.983–1.002)		
Bilirubin	0.253	0.983 (0.954–1.013)		
AST	0.229	1.000 (1.000–1.000)		
ALT	0.035	0.999 (0.999–0.999)	0.212	1.000 (0.999–1.000)
GGT	0.564	1.000 (0.999–1.001)		
ALP	0.005	1.002 (1.001–1.003)	0.003	1.002 (1.001–1.003)
LDH	0.618	1.000 (1.000–1.000)		
PCT	0.828	1.001 (0.994–1.008)		
CRP	0.220	1.001 (0.999–1.003)		
IL-6	0.008	1.000 (1.000–1.000)	0.031	1.000 (1.000–1.000)
Creatinine	0.997	1.000 (0.932–1.073)		
INR	0.526	0.940 (0.776–1.139)		

CI: confidence interval, BMD: bone mineral density, AST: aspartate transaminase, ALT: alanine transaminase, GGT: γ-Glutamyl transpeptidase, ALP: alkaline phosphatase, LDH: lactate dehydrogenase, PCT: procalcitonin, CRP: C-reactive protein, IL: interleukin, INR: International normalized ratio.

## Data Availability

The datasets used and/or analysed during the current study are available from the corresponding author on reasonable request.
